# Prevalence and Risk Factors of Soil-Transmitted Helminth Infections on Koh Yao Islands, Southern Thailand: A Community-Based Cross-Sectional Survey

**DOI:** 10.3390/ijerph23050595

**Published:** 2026-05-01

**Authors:** Chuchard Punsawad, Prasit Na-ek, Udomsak Narkkul, Chanakan Rattanaburi, Aunchisa Kongsuk, Tharathep Plub-on, Stephen J. Scholand, Nonthapan Phasuk

**Affiliations:** 1 School of Medicine, Walailak University, Nakhon Si Thammarat 80160, Thailand; chuchard.pu@wu.ac.th (C.P.); prasit.na@wu.ac.th (P.N.-e.); udomsak.na@wu.ac.th (U.N.); chanakan.rt@mail.wu.ac.th (C.R.); aunchisa.kn@mail.wu.ac.th (A.K.); tharathep.pl@mail.wu.ac.th (T.P.-o.); 2 Center of Excellence in Tropical Pathobiology, Walailak University, Nakhon Si Thammarat 80160, Thailand; 3 Department of Medicine, University of Arizona, Tucson, AZ 85724, USA; rabiesfreeworld@yahoo.com

**Keywords:** prevalence, risk factor, soil-transmitted helminths, Thailand

## Abstract

**Highlights:**

**Public health relevance—How does this work relate to a public health issue?**

- Soil-transmitted helminth (STH) infections are among the most prevalent neglected tropical diseases, particularly in rural areas in southern Thailand.
- This study investigates the prevalence of STH infections and identifies associated risk factors among adults in Koh Yao Islands, southern Thailand.

**Public health significance—Why is this work of significance to public health?**

- This study demonstrated a low prevalence of STH infections (2.49%) in Koh Yao Islands, southern Thailand, with hookworms and *T. trichiura* identified as the circulating species.
- It identified that all infected individuals had an educational level lower than a bachelor’s degree. In addition, the absence of hygienic toilet use at home was significantly associated with STH infections.

**Public health implications—What are the key implications or messages for practitioners, policy makers and/or researchers in public health?**

- This study demonstrated a low prevalence of STH infections in the Koh Yao Islands, southern Thailand. This suggests effective public health control of STH infections in the region.
- Sustained implementation of targeted public health measures, including periodic deworming and hygiene-focused health education, is essential to limit residual transmission.

**Abstract:**

Background: Soil-transmitted helminth (STH) infections are a recognized public health challenge, particularly in rural and island settings. Despite the implementation of national control programs, epidemiological data from geographically isolated communities remain limited. In this study, we aimed to determine the prevalence of STH infections and identify associated risk factors among adults in Koh Yao Islands, southern Thailand. Methods: A community-based cross-sectional survey was conducted in three subdistricts of Koh Yao, Phang Nga Province, from January to September 2024. Demographic data and information on potential risk factors were collected using structured questionnaires. Stool specimens were analyzed using the formalin–ethyl acetate concentration technique and the modified Kato–Katz method. Associations were assessed using univariate and multivariate logistic regression analyses. Results: A total of 241 adults participated in the study, with females accounting for 68.9% and males for 31.1%. The overall prevalence of STH infections was 2.49%, with hookworms and *Trichuris trichiura* identified as the predominant species. All infected individuals had an educational level lower than a bachelor’s degree. Univariate analysis showed that participants who did not use hygienic toilets at home had a significantly higher likelihood of developing STH than those who did (crude odds ratio = 46.80; 95% confidence interval [CI]: 2.55–859.00; *p* = 0.010). Multivariable logistic regression analysis confirmed that the absence of hygienic toilet use at home was independently associated with STH infection (adjusted odds ratio = 30.69; 95% CI: 1.17–804.65; *p* = 0.040). Conclusions: This study documents low overall prevalence of STH infections in the study area, with hookworms and *T. trichiura* as the predominant organisms. These findings support continued targeted public health measures, including periodic deworming and health education initiatives, to strengthen hygiene practices, particularly in high-risk populations. Future investigations should incorporate environmental assessments and longitudinal monitoring to evaluate the durability of current control strategies.

## 1. Introduction

Soil-transmitted helminth (STH) infections are among the most prevalent neglected tropical diseases globally, disproportionately affecting tropical regions characterized by limited sanitation and unsafe water supply [1,2]. In 2021, STH infections accounted for over 640 million cases and 1.3 million disability-adjusted life-years, reflecting a substantial global health burden [2]. Beyond direct clinical morbidity, such as impaired nutrition and reduced quality of life, these infections impose substantial socioeconomic costs through diminished labor productivity and increased healthcare expenditures [3,4,5]. Although the World Health Organization has set ambitious 2030 targets to eliminate STH-related morbidity, achieving these goals depends on the success of localized control programs [6,7].

Accumulating evidence from southern Thailand suggests that STH infections continue to circulate in certain communities despite ongoing control efforts. Previous studies have reported moderate to high prevalence rates among both adults and school-aged children, with hookworms consistently identified as the dominant species [8,9,10]. Recent national surveillance data further confirm that hookworm infection rates remain highest in the southern provinces of Thailand [11]. Persistent transmission of STH, driven by a complex interplay of environmental, behavioral, and structural factors, remains a considerable public health challenge in Thailand. Although control programs have historically targeted school-aged children, recent evidence suggests that older adults in rural areas carry a substantial and often overlooked burden of infection. STH prevalence as high as 15.7% has been documented among older adults in southern Thailand [8], while recent 2024 data reported a 9.72% infection rate among schoolchildren [12]. This elevated prevalence in the geriatric population likely reflects the cumulative lifetime exposure and the persistence of specific risk factors that bridge occupational and domestic life. Thailand’s current national strategies for controlling STH infections primarily utilize test-and-treat interventions and mass drug administration (MDA) with albendazole targeting schoolchildren. Nevertheless, adult and elderly populations remain vulnerable to infection and continue to represent significant at-risk groups [13].

Koh Yao District, an island archipelago in Phang Nga Province, serves as an important region for study. Its geographical isolation, dependence on boat transportation, and humid climate create distinct environments conducive to helminth transmission. While agriculture remains the primary livelihood, access to healthcare is more limited than on the mainland. Existing data from Koh Yao are largely limited to schoolchildren, among whom hookworms are the most commonly identified species [14]. Currently, epidemiological data on STH prevalence and behavioral risk factors among the adult population on these islands are lacking.

Regular monitoring of STH prevalence at the community level is essential to comprehensively understand local transmission dynamics and assess the effectiveness of existing control measures. In the absence of up-to-date data from the Koh Yao District, the status of STH infections and associated behavioral risk factors remains unclear. Accordingly, the objectives of this study include addressing the existing data gap by determining the prevalence of STH infections, and identifying specific socio-behavioral risk factors among adults in the Koh Yao District. This endeavor provides a framework for understanding targeted health strategies in isolated island communities.

## 2. Materials and Methods

### 2.1. Study Design and Population

A community-based cross-sectional investigation was conducted to assess the prevalence of STH infections and identify associated risk factors among adults living in Koh Yao District, Phang Nga Province, Thailand. The study population consisted of residents aged 18–80 years who had lived in the district for at least six months prior to data collection. According to the Bureau of Registration Administration, Koh Yao District has a total adult population of 8475 individuals, including 4290 males and 4185 females. Individuals were eligible for inclusion if they provided written informed consent, were willing to complete the questionnaire, or provided stool samples. Participants who were unable to participate owing to serious illness or declined sample collection were excluded from the study.

### 2.2. Study Setting

Koh Yao District is an island area situated in the Andaman Sea, encompassing approximately 137.6 km^2^. The district lies at approximately 11°37′ N latitude and 98°34′ E longitude and has a maximum elevation of approximately 261.6 m above sea level. Administratively, the district is subdivided into three subdistricts—Koh Yao Noi, Koh Yao Yai, and Phru Nai—comprising 18 villages (seven, four, and seven villages, respectively). The area experiences a tropical climate with substantial annual rainfall, averaging 1307 mm and reaching up to 1500 mm during peak periods. The healthcare infrastructure within the district includes one community hospital and three subdistrict health promotion hospitals, which serve as the primary providers of medical services. Access between Koh Yao District and the mainland is mainly facilitated by boat transportation.

### 2.3. Sample Size Calculation and Sampling

The sample size was determined using the single proportion population formula: N = Z^2^ P (1 − P)/D^2^. An expected prevalence of 18.1%, derived from previously reported national data [15], was used as the reference proportion. The precision level was set at 0.05, with a 95% confidence level. Based on these assumptions, the minimum required sample size was 227. To compensate for possible non-responses or incomplete data, an additional 10% was added, resulting in a target sample size of 252 individuals. Participants were recruited using random sampling. Sampling was conducted across the three subdistricts of Koh Yao District, with the number of participants from each subdistrict allocated proportionally to its population size to ensure adequate representation.

### 2.4. Questionnaire Survey

Data were collected using a structured questionnaire adapted from a previously validated instrument used in a study of soil-transmitted helminth infections in Southern Thailand [8]. The instrument was designed to obtain information on participants’ sociodemographic characteristics and health-related behaviors associated with STH infection. Behavioral items were organized into three main areas: practices associated with STH exposure, dietary habits, and behaviors related to the prevention and control of STH transmission at both household and community levels. The questionnaire’s content validity was confirmed by a panel of three experts in parasitology. The questionnaire demonstrated good internal reliability, with a Cronbach’s alpha coefficient of 0.827 [8], indicating acceptable consistency across items. Sociodemographic variables included age, sex, place of residence, and educational attainment. Information on potential exposure and protective factors, including the availability and use of household sanitation facilities, hand hygiene practices following defecation, and consumption of raw or inadequately washed vegetables, was also obtained from all participants.

### 2.5. Stool Collection and Parasitological Procedures

Participants were provided with clear instructions for self-collection of stool specimens and supplied with labeled, clean, and leak-proof containers. Each participant submitted a single fresh stool sample (approximately 2–10 g). Following collection, samples were transported without delay to field laboratories for parasitological assessment. All specimens were processed and examined using two standard diagnostic techniques: the formalin–ethyl acetate concentration method and the modified Kato–Katz procedure, as previously described. For the concentration procedure, approximately 1 g of stool was thoroughly mixed with 10% buffered formalin and filtered to remove large particles. Ethyl acetate was subsequently added, and the suspension was vigorously agitated before centrifugation at 3000 rpm for 5 min. Following centrifugation, the upper layers were carefully discarded, leaving the sediment, which was resuspended and examined microscopically for parasite eggs and other stages [14]. For the modified Kato–Katz technique was performed using a standardized template delivering approximately 41.7 mg of stool onto a glass slide. The sample was covered with a glycerol-based clearing solution–soaked cellophane strip and evenly spread to form a thick smear. After clearing at room temperature (30–60 min), slides were examined for helminth eggs [8]. All preparations were examined under a light microscope at 10× and 40× magnifications. To enhance diagnostic reliability, each specimen was independently assessed by two trained medical technologists who were blinded to participant information. Any discrepancies were reviewed and resolved by a senior examiner.

### 2.6. Ethical Considerations

This study was reviewed and approved by the Ethics Committee on Human Rights Related to Research Involving Human Subjects at the Walailak University (approval no. WUEC-23-347-01). Before participation, all participants received a detailed explanation of the study objectives and procedures, after which written informed consent was obtained. Participant confidentiality was strictly maintained throughout the study, and all data were anonymized and used solely for research purposes.

### 2.7. Statistical Analyses

Before data entry, all completed questionnaires and data collection forms were reviewed to ensure accuracy and internal consistency. Statistical analyses were performed using SPSS version 20.0 (IBM Corp., Armonk, NY, USA). Participant characteristics and health-related variables are summarized using descriptive statistics, including frequencies, means, and standard deviations, as appropriate. Associations between STH infection status and potential explanatory variables were examined using univariate logistic regression analysis. Variables demonstrating a *p*-value < 0.25 in the univariate models were subsequently included in multivariable logistic regression analyses to control for possible confounding effects. Statistical significance was defined as a two-sided *p*-value < 0.05.

## 3. Results

### 3.1. Demographic Characteristics

Of the 252 individuals initially approached, 241 completed the study, yielding a response rate of 95.6%. Females comprised 68.9% of the study population. Participants were predominantly middle-aged to older adults, with the largest proportion falling in the 51–65-year age group (47.3%), followed by those aged 36–50 years (23.7%). Half of the participants resided in the Phru Nai subdistrict (50.2%), whereas the remainder were distributed across the other two subdistricts. Most respondents were married (83.4%), and nearly all identified as Muslim (99.6%). A detailed summary of participants’ demographic characteristics is presented in Table 1.

### 3.2. Prevalence of STH Infections

Parasitological analysis of stool specimens from 241 participants identified STH infections in six individuals, corresponding to an overall prevalence of 2.49% (Table 2). A greater proportion of infections was observed among female participants, who accounted for 83.3% of positive cases, whereas males accounted for 16.7%. Hookworms and *Trichuris trichiura* were identified with equal frequencies, each accounting for three of the six infections detected (Table 2). All infected participants harbored a single parasite species. No other helminthic or protozoan species were found in this study. Age-specific analysis indicated variability in infection rates across age groups. The highest prevalence was observed among participants aged 18–35 years (5.6%). Lower prevalence was recorded in the 36–50 and 51–65 year age groups (1.8% each), whereas participants aged 66–80 years showed a prevalence of 2.9% (Table 2).

### 3.3. Health Behaviors Related to STH Infections

Table 3 presents the health behaviors related to STH infection among the participants. Overall, the participants reported high adherence to hygienic practices, including universal footwear use outdoors and hand washing after defecation. Nearly all participants reported washing their hands before meals (99.6%). Most individuals (85.9%) wore protective footwear during gardening or agricultural activities, and handwashing after soil contact was widely practiced (98.3%). Although potentially risky behaviors were reported at low frequencies, they remain epidemiologically relevant. A small proportion of participants occasionally consumed raw freshwater fish (0.8%) or handled pets regularly (22.8%). Although most respondents used hygienic toilets at home (98.8%), some reported intermittent defecation in pit latrines (8.7%) or on open ground (19.5%), which could contribute to environmental contamination. Regular nail trimming was reported by 74.4% of participants.

### 3.4. Associations of Risk Factors and STH Infections

Univariate analyses exploring demographic characteristics showed that STH infections were more frequently observed among females and participants aged < 66 years than among males and older individuals; however, neither sex nor age was significantly associated with infection status. All individuals diagnosed with STH had an educational level below a bachelor’s degree.

Behavioral and environmental factors were examined. Participants who did not use hygienic toilets at home had a significantly higher prevalence of STH infection than those who used hygienic sanitation facilities (crude odds ratio [COR] = 46.8; 95% confidence interval [CI]: 2.55–859.0; *p* = 0.010) (Table 4). Higher infection rates were also observed among individuals who did not regularly trim their nails and those who reported defecation in pit latrines or on open ground; however, these associations were not statistically significant. Regular consumption of fresh vegetables was inversely associated with STH infection and demonstrated a protective effect in univariate analysis (COR = 0.065; 95% CI: 0.01–0.74; *p* = 0.027) (Table 4).

In multivariable logistic regression analysis, lack of hygienic toilet use at home remained independently associated with STH infection after adjusting for potential confounders (adjusted odds ratio = 30.69; 95% CI: 1.17–804.65; *p* = 0.040) (Table 4).

## 4. Discussion

STH infections continue to warrant attention in communities in southern Thailand, although their burden has declined substantially in recent years. In the present study, the overall prevalence of STH infection was low (2.49%), lower than the estimates reported in recent national surveillance data [11]. Furthermore, the prevalence rate observed in this study was notably lower than recent reports among schoolchildren in southern Thailand, including those documented in Nakhon Si Thammarat Province (9.72%) [12] and the Koh Yao Islands (4.94%) [14]. This disparity aligns with the classic age-prevalence distribution of STH infections, in which children serve as the primary reservoir due to behavioral vulnerabilities, such as walking barefoot, inconsistent hand hygiene, and playing in contaminated soil. The low prevalence identified in the current study suggests a favorable epidemiological shift within the adult population. This likely results from the cumulative impact of long-standing control strategies, including expanded sanitation coverage, targeted health education, and periodic deworming programs [16]. The observed low prevalence aligns with the national goals for STH control and indicates sustained progress toward reducing community-level transmission. The study area has implemented basic sanitation infrastructure, including improved access to latrines and clean water in many households. In addition, periodic deworming programs, particularly those targeting school-aged children, have been conducted as part of national public health initiatives. Health education campaigns promoting personal hygiene, such as handwashing and safe food practices, may also have played a role in reducing transmission.

Regarding species composition, hookworms and *T. trichiura* were identified as the circulating STH species in this population, each accounting for half of the detected infections. This pattern is consistent with reports from several tropical settings, where hookworms remain the predominant STH, including Guinea-Bissau [17], Sierra Leone, southeast China [18], and Muleba district of Tanzania [19], particularly in agricultural communities. The persistence of hookworm transmission may be attributed to occupational exposure to contaminated soil and continued contact with moist environments favorable for larval development [20].

In contrast, *Ascaris lumbricoides* was not detected in this study, which differs from the findings reported in the Wolaita zone of Southern Ethiopia [21], Semarang, Central Java, Indonesia [22], and in the peri-urban areas of Jimma town, Oromia, Ethiopia [23], where this species remains highly prevalent. These discrepancies likely reflect ecological and environmental differences, including variations in soil characteristics, temperature, humidity, and sanitation infrastructure, all of which influence parasitic survival and transmission.

In the present study, STH infections were more frequently observed among female participants, particularly those aged 18–35 years. This age- and sex-specific pattern is consistent with earlier studies reporting a higher occurrence of STH infections among females and younger to middle-aged adults [17]. However, it should be noted that a larger number of females were recruited compared to males, which may have introduced a selection bias. The elevated risk among younger adults may reflect greater occupational exposure, as females in this community commonly participate in agricultural activities within a matrix social structure. This finding reinforces the concept of age-shift epidemiology in elimination settings, where the burden of infection is transferred from children to socioeconomically active adults [24]. Individuals in this age group are more likely to engage in outdoor activities and occupations such as agriculture, construction, or other labor-intensive work that increase exposure to contaminated soil. Additionally, differences in hygiene practices and the frequency of contact with potentially contaminated environments may contribute to the increased risk. Such activities are well recognized as potential sources of exposure to the infective stages of STH, particularly hookworms. Although most participants reported adopting protective behaviors, such as wearing boots during gardening, unsafe sanitation practices were still reported by a subset of the population, including occasional defecation in pit latrines or on open ground. These behaviors, even infrequently, may contribute to ongoing environmental contamination and sustain low-level transmission [25].

Farmers remain vulnerable owing to prolonged and repeated contact with soil and wastewater during daily work activities, which increases the likelihood of percutaneous or accidental exposure to infective larvae [26]. Similarly, studies examining risk factors in the region highlighted the inconsistent footwear use during farming as a major predictor of hookworm infections [27]. These findings highlight the need for sustained context-specific health education interventions. Programs should emphasize the consistent use of protective footwear, the promotion of hygienic sanitation facilities, and the reinforcement of personal hygiene practices. Such targeted interventions are essential for reducing soil contamination and preventing both initial infections and reinfections among adults in agricultural communities.

In the present study, all individuals diagnosed with STH had an educational level below a bachelor’s degree. This observation aligns with evidence from other endemic settings, where lower educational attainment has been associated with limited awareness and understanding of STH infections [28]. Insufficient knowledge about transmission routes, preventive measures, and health consequences may reduce engagement with effective control practices and undermine the success of intervention programs [29]. Multivariate analysis identified the lack of hygienic toilet use at home as an independent factor associated with STH. This finding is consistent with that of a previous study conducted in the Bijagos Islands, Guinea-Bissau [17]. The authors found that participants who did not regularly use hygienic sanitation facilities had a substantially higher likelihood of infection, highlighting sanitation as a critical determinant of ongoing transmission in this community [30]. Although the CI was wide, reflecting the low number of positive cases, the direction and magnitude of the association underscore the importance of safe sanitation practices in preventing STH transmission. Therefore, improving access to and the consistent use of hygienic toilets should remain a central component of local control strategies. Community-based initiatives, including support for household latrine construction and the expansion of affordable sanitation options, may help reduce environmental contamination. In parallel, health education efforts should emphasize the relationship between sanitation behaviors and helminth infection risk, encouraging sustained behavioral change to avoid open defecation. Incorporating behavior change communication and routine monitoring mechanisms could further strengthen the effectiveness of these interventions [14]. Future studies are warranted to explore the social, cultural, and structural barriers to hygienic toilet use and to inform the development of targeted, context-specific sanitation programs.

This study has several limitations that should be considered when interpreting the findings. First, STH prevalence and infection intensity were estimated using a single stool specimen per participant. Given the known day-to-day variation in helminth egg excretion, this approach may have resulted in decreased diagnostic sensitivity, likely underestimating the true prevalence of STH infections in the community. Second, although we employed dual parasitological techniques (FECT and modified Kato–Katz) to maximize the diagnostic yield from these single specimens, traditional microscopic methods are inherently less sensitive than modern molecular tools. Recent literature emphasizes the shift toward quantitative PCR (qPCR), which offers superior species differentiation and improved detection of low-intensity infections [31]. Third, information on sociodemographic characteristics and behavioral risk factors was self-reported and is therefore subject to recall and social desirability bias, potentially resulting in the misclassification of exposure variables; for instance, social desirability could lead participants to over-report positive hygiene practices, such as handwashing or footwear use, thereby potentially masking their protective effects. Similarly, recall bias regarding past exposures may have introduced non-differential misclassification, which typically biases results toward the null. Despite these limitations, this study provided valuable insights into the current epidemiological status of STH infection in a geographically isolated community.

## 5. Conclusions

This study demonstrated a low prevalence of STH infections in the study community, with hookworms and *T. trichiura* identified as the circulating species. Infections were observed exclusively among individuals with educational attainment below the bachelor’s level, underscoring the role of education in STH prevention. Sustained implementation of targeted public health measures, including periodic deworming and hygiene-focused health education, is essential to limit residual transmission. Future investigations should incorporate environmental assessments and longitudinal monitoring to evaluate the durability of current control strategies. In addition, studies examining community knowledge, attitudes, and practices related to STH infection would provide valuable evidence to strengthen preventive and control efforts.

## Figures and Tables

**Table 1:** Baseline characteristics of participants from Koh Yao District, Phang Nga Province (*n* = 241).

Characteristics		Number	Percentage
Sex			
	Male	75	31.1
	Female	166	68.9
Age			
	18–35	36	14.9
	36–50	57	23.7
	51–65	114	47.3
	66–80	34	14.1
Setting			
	Koh Yao Noi	46	19.09
	Koh Yao Yai	74	30.7
	Phru Nai	121	50.21
Status			
	Single	22	9.13
	Married	201	83.4
	Divorced	18	7.47
Religion			
	Muslim	240	99.59
	Christian	1	0.41

**Table 2:** Distribution of soil-transmitted helminth (STH) infections by area, sex, parasite species, and age group among study participants (*n* = 241).

Variable	Category	Total Examined, *n* (%)	Positive Cases, *n* (%)
Study area	Koh Yao Noi	46 (19.09)	2 (4.35)
	Koh Yao Yai	74 (30.71)	1 (1.35)
	Phru Nai	121 (50.21)	3 (2.48)
Sex	Male	75 (31.12)	1 (1.33)
	Female	166 (68.88)	5 (3.01)
Parasite species	Hookworm	–	3 (50.0)
	*Trichuris trichiura*	–	3 (50.0)
Age group (years)	18–35	36 (14.94)	2 (5.56)
	36–50	57 (23.65)	1 (1.75)
	51–65	114 (47.30)	2 (1.75)
	66–80	34 (14.11)	1 (2.94)
Overall	Total	241 (100.00)	6 (2.49)

**Table 3:** Health behaviors related to soil-transmitted helminth (STH) infections among participants in Koh Yao, Phang Nga Province, Thailand (*n* = 241).

Behaviors	Practice (Number, %)		
	Always	Sometimes	Never
1. Wears shoes when going outside	241 (100.00)	0 (0.00)	0 (0.00)
2. Wears sneakers/boots while gardening	207 (85.80)	27 (11.20)	7 (3.00)
3. Eats raw/undercooked freshwater fish	2 (0.83)	2 (0.83)	237 (98.34)
4. Eats raw/undercooked pork or beef	0 (0.00)	0 (0.00)	241 (100.00)
5. Eats fresh vegetables	115 (47.72)	122 (50.62)	4 (1.66)
6. Washes hands before eating	240 (99.59)	1 (0.41)	0 (0.00)
7. Washes hands after defecation	241 (100.00)	0 (0.00)	0 (0.00)
8. Washes hands after soil-related activities	237 (98.34)	4 (1.66)	0 (0.00)
9. Frequency of pet handling per week	55 (22.82)	105 (43.57)	81 (33.61)
10. Uses hygienic toilet at home	239 (98.17)	2 (0.83)	0 (0.00)
11. Defecates in hole, ground, or garden	21 (8.72)	47 (19.50)	173 (71.78)
12. Regular weekly nail trimming	180 (74.68)	58 (24.10)	3 (1.22)

**Table 4:** Risk factors associated with soil-transmitted helminth (STH) infections among participants in Ko Yao District, Phang Nga Province.

Characteristics		*n*	STH	OR (95% CI)	*p*-Value	AOR * (95% CI)	*p*-Value
Sex							
	Male	75	1 (1.33)	Ref.		-	-
	Female	166	5 (3.01)	2.30 (0.26–20.02)	0.451		
Age group							
	≤65	207	5 (2.42)	Ref.		-	-
	>65	34	1 (2.94)	1.22 (0.14–10.81)	0.856		
Education level							
	<Higher education	223	6 (2.69)	-	-	-	-
	≥Higher education	18	0 (0)	-	-	-	-
Having pets							
	No	108	3 (2.78)	Ref.		-	-
	Yes	133	3 (2.26)	0.81 (0.16–4.09)	0.796	-	-
Wearing boots when gardening							
	No	34	1 (2.94)	0.82 (0.09–7.21)	0.856	-	-
	Yes	207	5 (2.42)	Ref			
Eating fresh vegetable							
	No	4	1 (25.00)	Ref		Ref	
	Yes	237	5 (2.11)	0.07 (0.01–0.74)	0.027	0.02 (0.00–0.37)	0.008
Cut nails							
	Rarely/Sometimes	61	3 (4.92)	3.05 (0.60–15.54)	0.179	3.89 (0.66–15.54)	0.164
	Always	180	3 (1.67)	Ref		Ref	
Defecate in toilet at home							
	No	2	1 (50.0)	46.80 (2.55–859.00)	0.010	30.69 (1.17–804.65)	0.040
	Yes	239	5 (2.09)	Ref		Ref	
Defecate on ground							
	No	173	3 (1.73)	2.62 (0.52–13.29)	0.246	2.29 (0.31–16.88)	0.418
	Yes	68	3 (4.41)	Ref		Ref	

* Significant association. COR, crude odds ratio; AOR, adjusted odds ratio; CI, confidence interval.

## Data Availability

The data supporting the findings of this manuscript are available from the corresponding authors upon reasonable request.
